# Supplementary material for: Non-viral generation of transgenic non-human primates via the piggyBac transposon system
Source: Nat Commun. 2025 Mar 24;16:2179. doi: 10.1038/s41467-025-57365-w (PMC11933304; doi:10.1038/s41467-025-57365-w)
Supplement: Supplementary file 1 — Supplementary Information [file 41467_2025_57365_MOESM1_ESM.pdf]

Non-viral generation of transgenic non-human primates via the piggyBac transposon system

Nakaya et al.

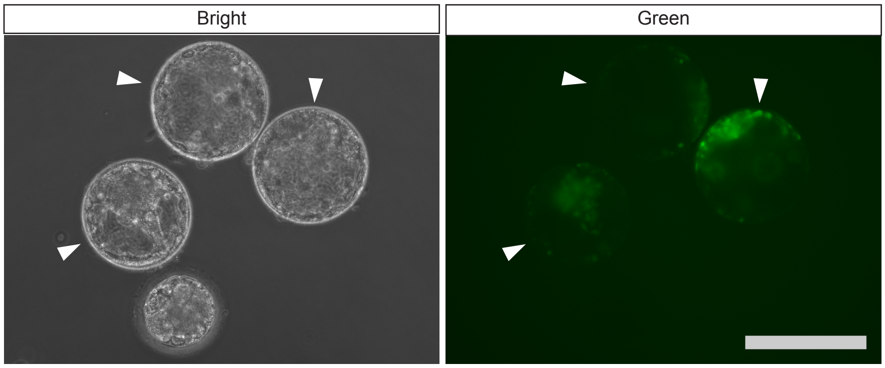

Supplementary Figure 1. Expression of autofluorescence in monkey blastocyst embryos. Green fluorescence in the wild-type monkey blastocyst embryos. Scale bar, 200 μm.

a

PB vector	PBase	Injected	Survived	Blastocysts (%/Survived)
0	0	5	5	5 (100)
1	0	11	11	4 (36.4)
1	50	12	12	9 (75.0)
5	0	10	10	6 (60.0)
5	50	21	19	2 (10.5)
10	0	10	7	6 (85.7)
10	50	20	20	2 (10.0)
30	0	16	15	1 (6.7)
30	50	22	17	0 (0)

b

PB vector	PBase	Injected	Survived	Blastocysts (%/Survived)
0	0	13	10	9 (90.0)
1	0	15	14	12 (85.7)
1	50	13	10	5 (50.0)
5	0	25	21	4 (19.0)
5	50	15	13	3 (23.1)
10	0	42	32	7 (21.9)
10	50	16	11	4 (36.4)
30	0	14	11	4 (36.4)
30	50	28	25	4 (16.0)

Supplementary Figure 2. Developmental rates of embryos after PN injection and co-injection. a Developmental rates of embryos after PN injection. The units of concentration for both the PB vector and PBase are ng/μL. **b** Developmental rates of embryos after co-injection.

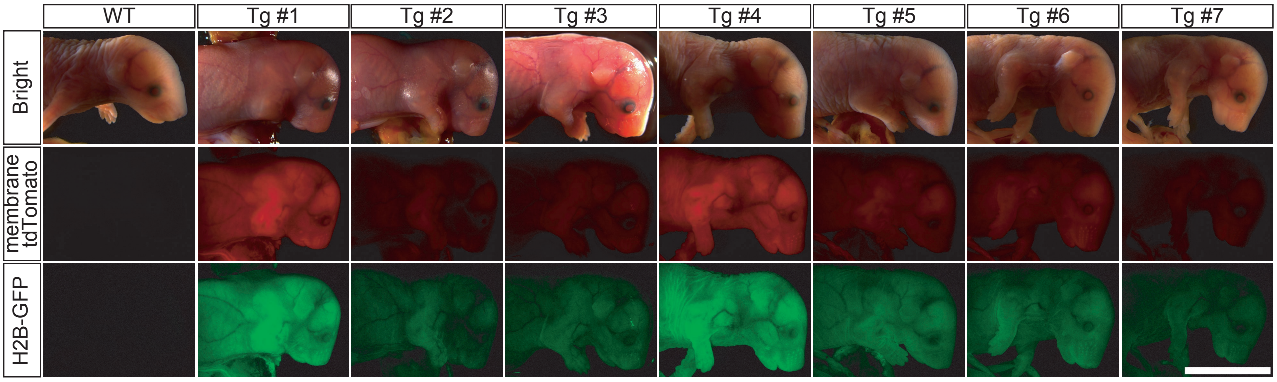

Supplementary Figure 3. Generation of transgenic mice after embryo transfer of co-injection. Expressions of the fluorescence reporters in the transgenic mice. Scale bar, 10 mm.

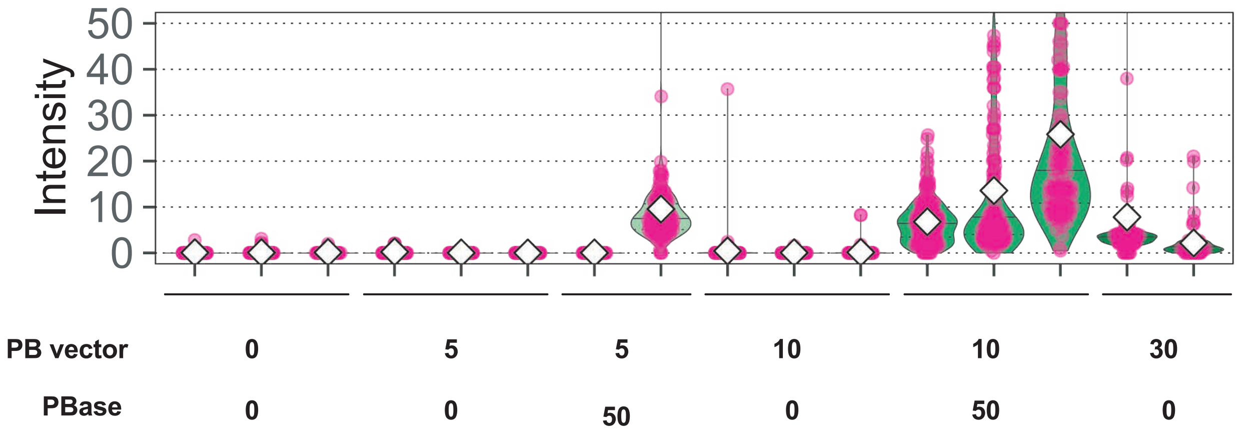

Supplementary Figure 4. Expressions of GFP in each monkey co-injection embryo. Violin plots of GFP intensities in each nucleus of each co-injected embryo. The top and bottom edges of the violins indicate the maximum and minimum values, respectively; the center lines indicate the medians; and the dotted lines indicate the first and third quartiles, respectively; the diamonds are the mean values. “PB” indicates piggyBac. The numbers below each panel are given in ng/μL. Source data are provided as a Source Data file.

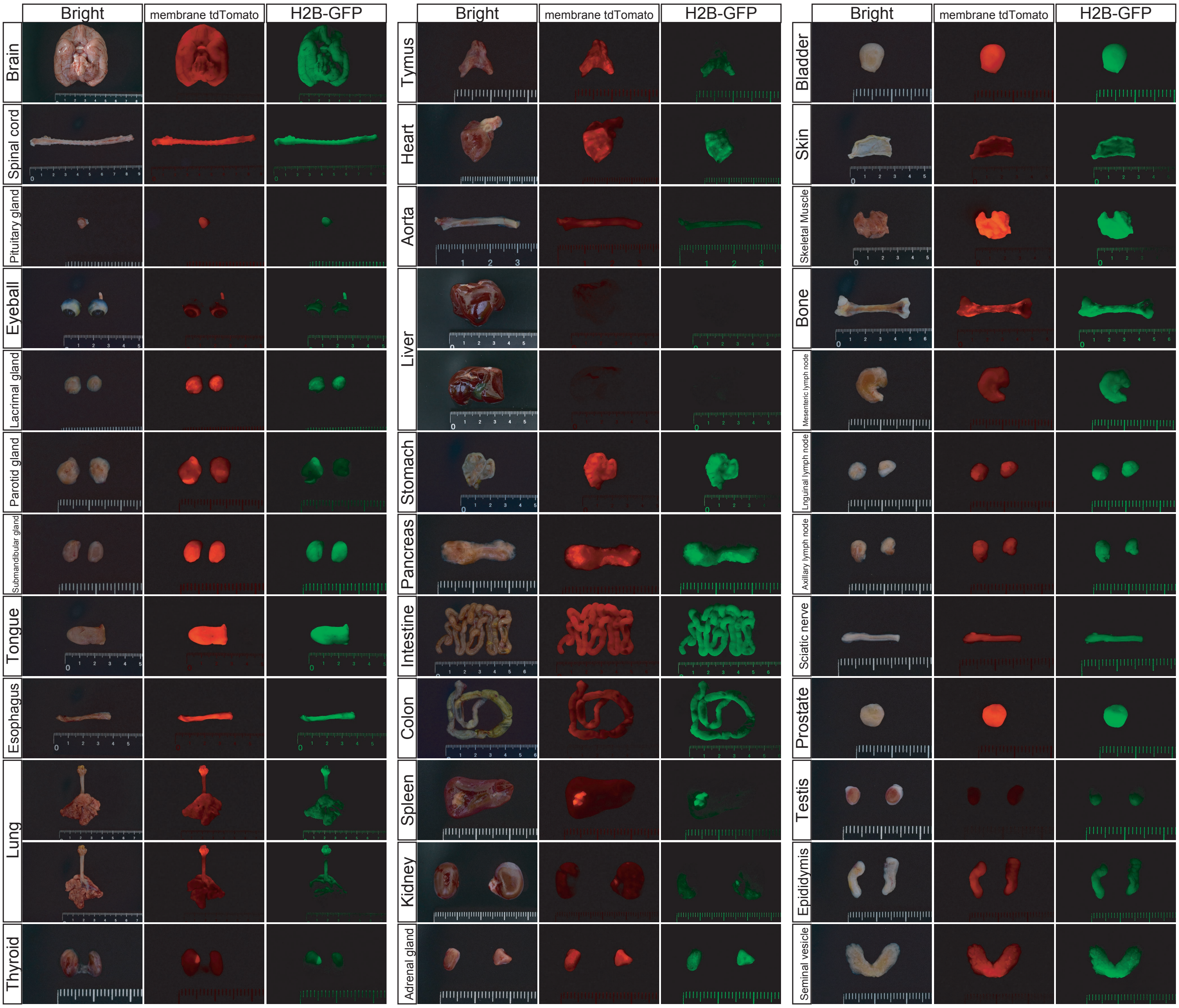

Supplementary Figure 5. Expression of fluorescence reporters in the organs from the delivered monkey (#1). Expression of fluorescence reporters in the transgenic monkey.

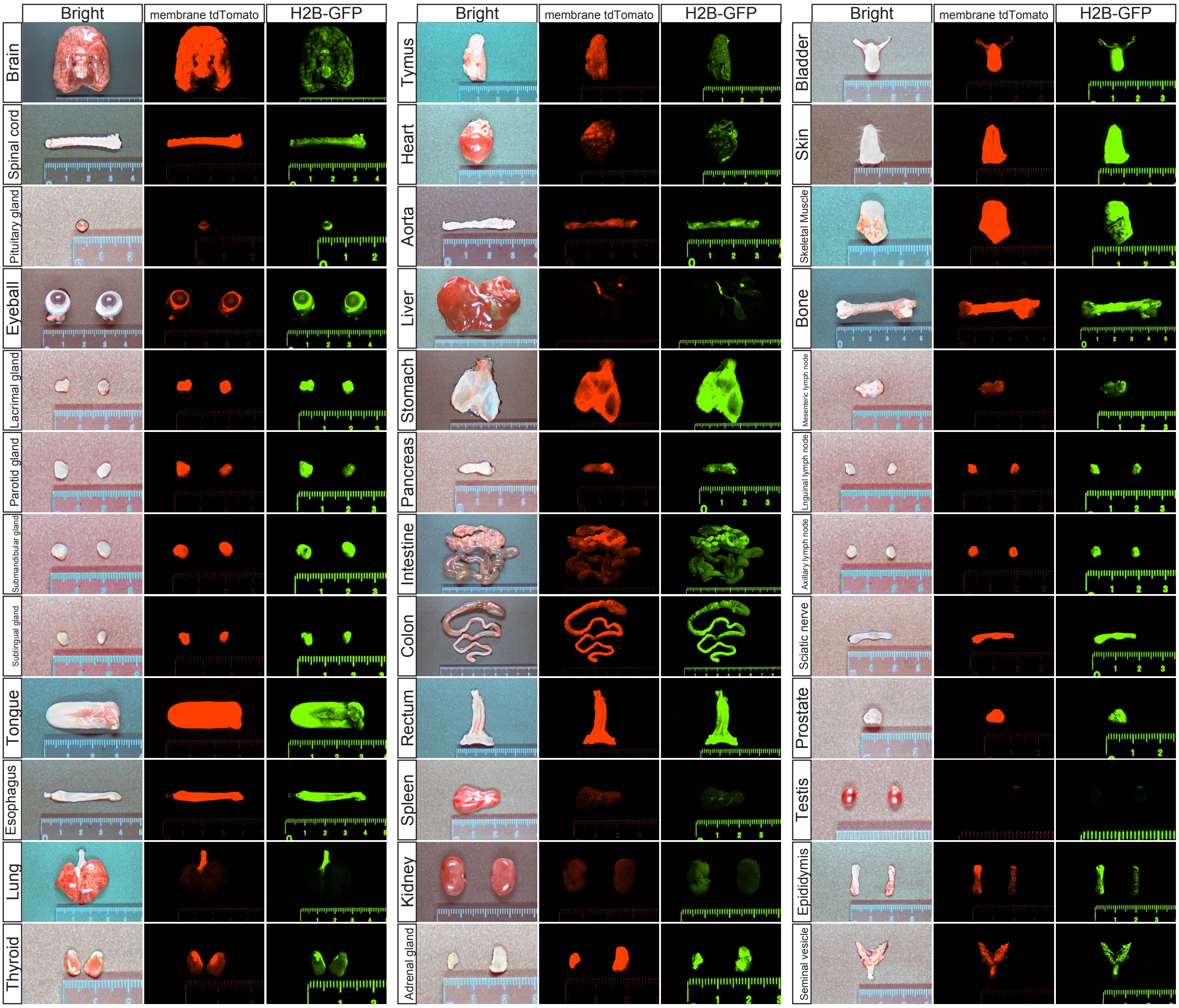

Supplementary Figure 6. Expression of fluorescence reporters in the organs from the stillborn monkey (#4). Expression of fluorescence reporters in the transgenic monkey.

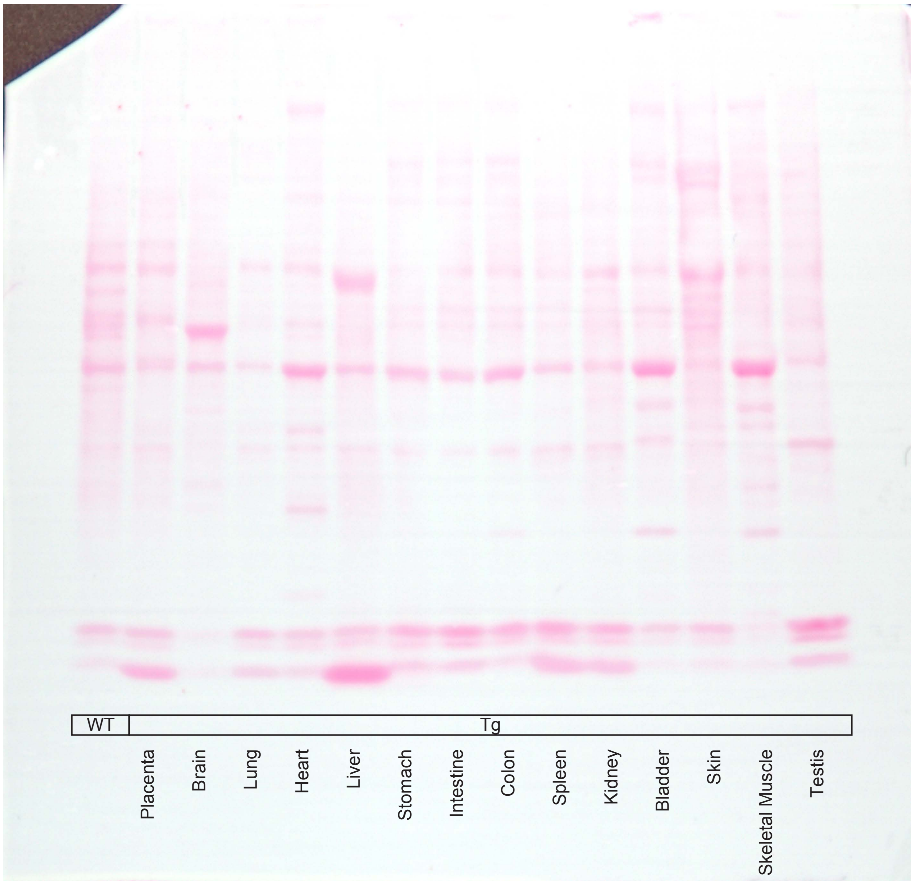

Supplementary Figure 7. Ponceau S staining for total protein detection. Ponceau S staining of the membrane used for Western blotting.

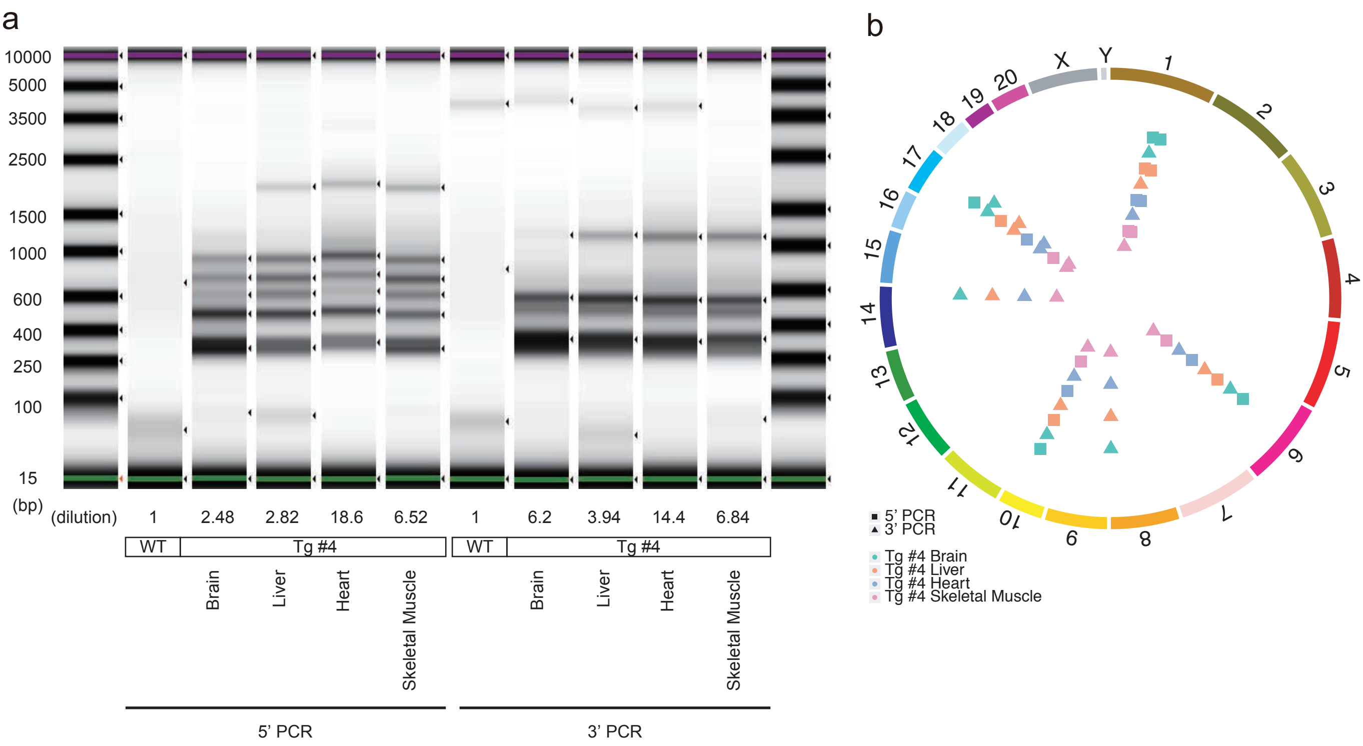

Supplementary Figure 8. Identification of the transgene insertion sites in the tissues of transgenic monkeys. a Inverse PCR for detection of transgene insertions in each tissue. **b** NGS sequencing for mapping of the insertion sites. Source data are provided as a Source Data file.

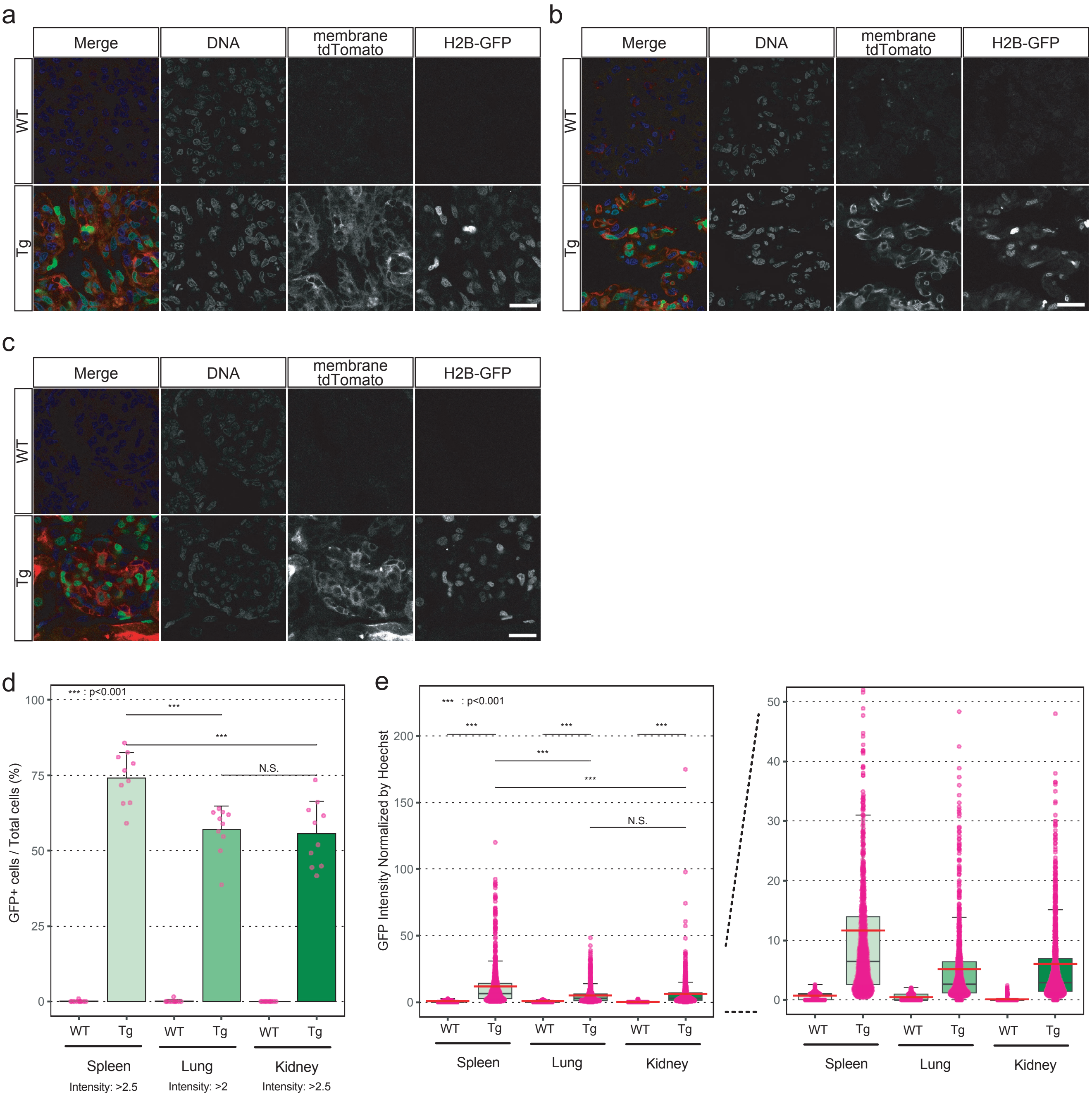

Supplementary Figure 9. Immunofluorescence analysis for the expression of transgenes. a–c Expression of fluorescence reporters in the spleen, lung and kidney, respectively. Scale bar, 20 μm. **d** Bar graph showing the GFP-positive cell rates based on the analysis of 10 fields per sample. Error bars, mean values + s.d.; n = 10 fields in each sample. One-way ANOVA and Tukey-Kramer post-hoc contrasts were used for the comparisons. *** P < 0.001; N.S. not significant. **e** Box plots of GFP intensities in each nucleus of the images. The top and bottom edges of boxes indicate the first and third quartiles, respectively; the center lines indicate the medians; the ends of whiskers indicate the maximum and minimum values within 1.5 times the interquartile range; and the red lines indicate the means. n = 10 fields in each sample. One-way ANOVA and Tukey-Kramer post-hoc contrasts were used for the comparisons. *** P < 0.001; N.S. not significant. Source data and P values are provided as a Source Data file.

Supplementary Table 1. Oligonucleotides used in this study.

Names	Forward	Reverse	For
XhoI_membrane_tdTomato_F, H2B_GFP_NotI_R	ctcgagagaattagcttgattcgagcctctagagc	gcggccgcTCACCTATTATCACGGCCGCC	PB-CAG-membrane tdTomato-2A-H2B-GFP cloning
KpnI_hyPBase_F, NotI_R	GGTACCGAGCTCGGATCCACTAGTAAC	gcggccgcTCATCAGAAACAGC	pcDNA3.1-hyPBase-poly(A83) cloning
EGFP_geno_F, R	GACCCTGAAGTTCATCTGCACCACCG	CTTGTACAGCTCGTCCATGCCGTGAG	Genotyping (EGFP)
tdTomato_geno_F, R	GCAAGGGCGAGGAGGTCATCAAAGAG	TGACGGCCATGTTGTTGTCCTCGGAG	Genotyping (tdTomato)
ROSA26_geno_F, R	TCCTGAAAAGGGTATAAACGTGGAGTAGGC	GACCAGCAATAACGTGTAGAATGCCATGAG	Genotyping (Mouse ROSA26)
beta-actin_geno_F, R	CCACACCTTCTACAATGAGCTGCGTG	GGCTCCACTTAGACCTACTGTGCATCTAC	Genotyping (Mouse beta actin)
Cyn_ZFX_F,R	ATTCCAGGCAGTACCAAACAG	CCATCAGGGCCAATAATTATT	Genotyping (Sex determination)
Cyn_beta-globin_genome_F, R	AAGGTGAACGTGGATGAAGTTGGTGG	TGTCTCTTCCCCATTCTAAACTGTACCCTG	Genotyping (Monkey beta globin)
EGFP_F, R	CCACATGAAGCAGCACGAC	TGCGCTCCTGGACGTAG	ddPCR (EGFP)

EGFP_probe	TTCTTCAAGTCCGCCATGCCCG		ddPCR (EGFP Probe)
tdTomato_F, R	CCCACAACGAGGACTACAC	CTTTGATGACGGCCATGTTG	ddPCR (tdTomato)
tdTomato_probe	CATCGTGGAACAGTACGAGCGCT		ddPCR (tdTomato probe)
5'_TR_F, R	TTAACCCTAGAAAGATAGTCTGC	AAGCGGCGACTGAGATG	ddPCR (5' TR)
5'_TR_probe	CTAAATGCACAGCGACGGATTCGC		ddPCR (5' TR probe)
Cyn_beta-globin_F, R	GGTATCAAGGTTACAAGACATGC	CAATAGGCAGAGAGAGTCAGTG	ddPCR (Monkey beta globin)
Cyn_beta-globin_probe	AGGAGGCAAATAGAAGCTGGGCAT		ddPCR (Monkey beta globin probe)
qTg_EGFP_F, R	TGAGCAAAGACCCCAACGAG	TTGTACAGCTCGTCCATGCC	qPCR (EGFP)
qTotal_Cyn_GAPDH	TTCAACAGCGACACCCACTC	GTTGCTGTAGCCAAATTCGTTG	qPCR (Gapdh)
PB5-fwd, rev	ACACTCTTTCCCTACACGACGCTCTTCCGATCTAGTTCATGCGCTTCAAGGTG	CAGACGTGTGCTCTTCCGATCTTCAAGAATGCATGCGTCAAT	For Inverse PCR (1st 5')
PB3-fwd, rev	ACACTCTTTCCCTACACGACGCTCTTCCGATCTGCAACTAGAAGGCACAGTCG	CAGACGTGTGCTCTTCCGATCTCCGATAAAACACATGCGTCA	For Inverse PCR (1st 3')

2nd-Rd1-1	AATGATACGGCGACCACCGAGATC TACACCTCTCTATACACTCTTTCCC TACACGACGCTCT		For Inverse PCR (2nd)
2nd-Rd1-2	AATGATACGGCGACCACCGAGATC TACACTATCCTCTACACTCTTTCCC TACACGACGCTCT		For Inverse PCR (2nd)
2nd-Rd1-3	AATGATACGGCGACCACCGAGATC TACACGTAAGGAGACACTCTTTCC CTACACGACGCTCT		For Inverse PCR (2nd)
2nd-Rd1-4	AATGATACGGCGACCACCGAGATC TACACACTGCATAACACTCTTTCCC TACACGACGCTCT		For Inverse PCR (2nd)
2nd-Rd1-5	AATGATACGGCGACCACCGAGATC TACACAAGGAGTAACACTCTTTCC CTACACGACGCTCT		For Inverse PCR (2nd)
2nd-Rd1-6	AATGATACGGCGACCACCGAGATC TACACCTAAGCCTACACTCTTTCCC TACACGACGCTCT		For Inverse PCR (2nd)
2nd-Rd1-7	AATGATACGGCGACCACCGAGATC TACACCGTCTAATACACTCTTTCCC TACACGACGCTCT		For Inverse PCR (2nd)
2nd-Rd1-8	AATGATACGGCGACCACCGAGATC TACACTCTCTCCGACACTCTTTCCC TACACGACGCTCT		For Inverse PCR (2nd)
2nd-Rd2-1	CAAGCAGAAGACGGCATACGAGAT GATCTGGTGACTGGAGTTCAGACG TGTGCTCTTCCGATCT		For Inverse PCR (2nd)
2nd-Rd2-2	CAAGCAGAAGACGGCATACGAGAT TCAAGTGTGACTGGAGTTCAGACG TGTGCTCTTCCGATCT		For Inverse PCR (2nd)

2nd-Rd2-3	CAAGCAGAAGACGGCATACGAGATCTGATCGTGACTGGAGTTCAGACGTGTGCTCTTCCGATCT		For Inverse PCR (2nd)
2nd-Rd2-4	CAAGCAGAAGACGGCATACGAGATAAGCTAGTGACTGGAGTTCAGACGTGTGCTCTTCCGATCT		For Inverse PCR (2nd)
2nd-Rd2-5	CAAGCAGAAGACGGCATACGAGATGTAGCCGTGACTGGAGTTCAGACGTGTGCTCTTCCGATCT		For Inverse PCR (2nd)
2nd-Rd2-6	CAAGCAGAAGACGGCATACGAGATTACAAGGTGACTGGAGTTCAGACGTGTGCTCTTCCGATCT		For Inverse PCR (2nd)
2nd-Rd2-7	CAAGCAGAAGACGGCATACGAGATTTGACTGTGACTGGAGTTCAGACGTGTGCTCTTCCGATCT		For Inverse PCR (2nd)
2nd-Rd2-8	CAAGCAGAAGACGGCATACGAGATGGAACTGTGACTGGAGTTCAGACGTGTGCTCTTCCGATCT		For Inverse PCR (2nd)
CAG_promoter_Bisulfite	GTAGTTATTGTTTTTTATGGTAA	AAAATAATAAAACAACACAATAACCAACAC	For Bisulfite PCR
