# Supplementary material for: Non-viral generation of transgenic non-human primates via the piggyBac transposon system
Source: Nat Commun. 2025 Mar 24;16:2179. doi: 10.1038/s41467-025-57365-w (PMC11933304; doi:10.1038/s41467-025-57365-w)
Supplement: Supplementary file 2 — Reporting Summary [file 41467_2025_57365_MOESM2_ESM.pdf]

nature portfolio

Corresponding author(s): Tomoyuki Tsukiyama

Last updated by author(s): Feb 12, 2025

Reporting Summary

Nature Portfolio wishes to improve the reproducibility of the work that we publish. This form provides structure for consistency and transparency in reporting. For further information on Nature Portfolio policies, see our Editorial Policies and the Editorial Policy Checklist.

Statistics

For all statistical analyses, confirm that the following items are present in the figure legend, table legend, main text, or Methods section.

n/a	Confirmed	
☐	☒	The exact sample size (*n*) for each experimental group/condition, given as a discrete number and unit of measurement
☐	☒	A statement on whether measurements were taken from distinct samples or whether the same sample was measured repeatedly
☐	☒	The statistical test(s) used AND whether they are one- or two-sided<br>*Only common tests should be described solely by name; describe more complex techniques in the Methods section.*
☒	☐	A description of all covariates tested
☐	☒	A description of any assumptions or corrections, such as tests of normality and adjustment for multiple comparisons
☐	☒	A full description of the statistical parameters including central tendency (e.g. means) or other basic estimates (e.g. regression coefficient) AND variation (e.g. standard deviation) or associated estimates of uncertainty (e.g. confidence intervals)
☐	☒	For null hypothesis testing, the test statistic (e.g. *F*, *t*, *r*) with confidence intervals, effect sizes, degrees of freedom and *P* value noted<br>*Give P values as exact values whenever suitable.*
☒	☐	For Bayesian analysis, information on the choice of priors and Markov chain Monte Carlo settings
☒	☐	For hierarchical and complex designs, identification of the appropriate level for tests and full reporting of outcomes
☒	☐	Estimates of effect sizes (e.g. Cohen's *d*, Pearson's *r*), indicating how they were calculated

Our web collection on statistics for biologists contains articles on many of the points above.

Software and code

Policy information about availability of computer code

Data collection	BD CellQuest Pro for FACS data collection.
Data analysis	FlowJo V9 was used for FACS data analysis.<br>R was used for statistical analysis.<br>ImageJ was used for image analysis.

For manuscripts utilizing custom algorithms or software that are central to the research but not yet described in published literature, software must be made available to editors and reviewers. We strongly encourage code deposition in a community repository (e.g. GitHub). See the Nature Portfolio guidelines for submitting code & software for further information.

Data

Policy information about availability of data

All manuscripts must include a data availability statement. This statement should provide the following information, where applicable:

- Accession codes, unique identifiers, or web links for publicly available datasets
- A description of any restrictions on data availability
- For clinical datasets or third party data, please ensure that the statement adheres to our policy

The source data underlying Figs. 1e–h, 2d–e, 3b–c, 4b, 5a–c, 6b–d, 7e–f, 8d, 8f–h, 9b–c and Supplementary Figs. 4, 8b, and 9d–e are provided as a Source Data file. The accession number for the sequencing data generated in this study is DRA019092.

Research involving human participants, their data, or biological material

Policy information about studies with human participants or human data. See also policy information about sex, gender (identity/presentation), and sexual orientation and race, ethnicity and racism.

Reporting on sex and gender	n/a
Reporting on race, ethnicity, or other socially relevant groupings	n/a
Population characteristics	n/a
Recruitment	n/a
Ethics oversight	n/a

Note that full information on the approval of the study protocol must also be provided in the manuscript.

Field-specific reporting

Please select the one below that is the best fit for your research. If you are not sure, read the appropriate sections before making your selection.

☒ Life sciences ☐ Behavioural & social sciences ☐ Ecological, evolutionary & environmental sciences

For a reference copy of the document with all sections, see nature.com/documents/nr-reporting-summary-flat.pdf

Life sciences study design

All studies must disclose on these points even when the disclosure is negative.

Sample size	The minimum number of animals necessary to achieve the scientific objectives was used because of the ethical reason.
Data exclusions	No data were excluded.
Replication	Some experiments producing the key data were repeated by different co-authors.
Randomization	Multiple animals were used for each biological replicate.
Blinding	All data were checked by multiple individuals who didn't know the genotype of animals.

Reporting for specific materials, systems and methods

We require information from authors about some types of materials, experimental systems and methods used in many studies. Here, indicate whether each material, system or method listed is relevant to your study. If you are not sure if a list item applies to your research, read the appropriate section before selecting a response.

Materials & experimental systems

n/a	Involved in the study	
☐	☒	Antibodies
☒	☐	Eukaryotic cell lines
☒	☐	Palaeontology and archaeology
☐	☒	Animals and other organisms
☒	☐	Clinical data
☒	☐	Dual use research of concern
☒	☐	Plants

Methods

n/a	Involved in the study	
☒	☐	ChIP-seq
☐	☒	Flow cytometry
☒	☐	MRI-based neuroimaging

Antibodies

Antibodies used	The antibodies used in the immunostaining were anti-GFP, Alexa Fluor 488–conjugated (A21311, 1:300 dilution; Thermo Fisher Scientific), anti-RFP (5F8) (PGI-5F8, 1:1000 dilution; ChromoTek), and anti-DDX4 (ab27591, 1:100 dilution; Abcom).<br>The antibodies used in the Western blotting were anti-GFP, Alexa Fluor 488–conjugated (A21311, 1:2000 dilution; Thermo Fisher Scientific), HRP-conjugated secondary antibodies (65-6120, 1:2000 dilution; Thermo Fisher Scientific), anti-beta-actin pAb-HRP-DirectT (PM053-7, 1:2000 dilution; MBL) and anti-GAPDH pAb-HRP-DirectT (M171-7, 1:8000 dilution; MBL).

Validation	We performed the staining with negative control.

Animals and other research organisms

Policy information about studies involving animals; ARRIVE guidelines recommended for reporting animal research, and Sex and Gender in Research

Laboratory animals	Cynomolgus monkeys
Wild animals	This study did not involve wild animals.
Reporting on sex	This study did not apply to only one sex.
Field-collected samples	This study did not involve samples collected from the field.
Ethics oversight	All animal experimental procedures were approved by the Animal Care and Use Committee of Shiga University of Medical Science (approval numbers: 2019-7-12, 2021-8-3, 2022-6-13 and 2024-1-3).

Note that full information on the approval of the study protocol must also be provided in the manuscript.

Plants

Seed stocks	n/a
Novel plant genotypes	n/a
Authentication	n/a

Flow Cytometry

Plots

Confirm that:

- [x] The axis labels state the marker and fluorochrome used (e.g. CD4-FITC).
- [x] The axis scales are clearly visible. Include numbers along axes only for bottom left plot of group (a 'group' is an analysis of identical markers).
- [x] All plots are contour plots with outliers or pseudocolor plots.
- [x] A numerical value for number of cells or percentage (with statistics) is provided.

Methodology

Sample preparation	Cells were dissociated and filtered into single cells with trypsin.
Instrument	BD FACS Calibur
Software	BD CellQuest Pro for data collection;<br>FlowJo V9 for data analysis
Cell population abundance	The purity of the samples was determined by PCR.
Gating strategy	To defined the boundaries, nonstaining samples were used to set the gate.

- [x] Tick this box to confirm that a figure exemplifying the gating strategy is provided in the Supplementary Information.
